# Seasonal and Daily Xylem Radius Variations in Scots Pine Are Closely Linked to Environmental Factors Affecting Transpiration

**DOI:** 10.3390/biology12091251

**Published:** 2023-09-18

**Authors:** Walter Oberhuber, Andreas Gruber, Gerhard Wieser

**Affiliations:** Department of Botany, University of Innsbruck, Sternwartestrasse 15, A-6020 Innsbruck, Austria; andreas.gruber@uibk.ac.at (A.G.); gerhard.wieser@uibk.ac.at (G.W.)

**Keywords:** atmospheric water demand, point dendrometer, xylem–phloem hydraulic coupling, Scots pine, stem radius changes, xylem radius variation

## Abstract

**Simple Summary:**

Combined measurements of radial variations of the water and carbon conducting tissue (i.e., the xylem and phloem, respectively) are valuable in order to study water exchange between tissues and water shortages in tree stems. In this study, we determined diurnal and seasonal radius variations of the xylem and inner bark (i.e., the tissue including phloem, parenchyma and meristem cells) of mature Scots pine trees (*Pinus sylvestris*) at a drought prone site, using devices that continuously record variations in stem radius with high resolution, so-called point dendrometers. The results of our study revealed that daily and seasonal radial variations of the xylem and inner bark were closely linked, indicating intensive water exchange between these tissues. A comparison of radial fluctuations in the xylem (XRV) with environmental variables affecting transpiration revealed a close coupling of XRV to vapor pressure deficit (VPD), i.e., the drying power of the air and air temperature, pointing to a strong dependence of stem water status on changes in atmospheric conditions. Because VPD increases exponentially with the increase in temperature and thus causes the water content in the stem to decrease especially under drought conditions, tree growth and mortality will be increasingly affected by climate warming.

**Abstract:**

Seasonal and daily radius variations in the xylem (XRV) and inner bark (IBV) of mature Scots pine trees (*Pinus sylvestris*) were determined during April 2019–October 2021 at a drought-prone inner alpine site (*c*. 750 m asl; Tyrol, Austria) by applying point dendrometers. XRVs were also related to environmental factors to evaluate the drivers of XRV during the growing season. XRV records revealed that the xylem width (i) started to shrink around the onset of radial stem growth in April, (ii) consistently decreased by *c*. 50 µm at the time when air temperature (T) and vapor pressure deficit (VPD) reached their maximum in late June through mid-July, and (iii) recovered until November/December. Although in daily cycles of radius variations XRV preceded IBV by about two hours and the daily amplitude of XRV was about 1/10 that of IBV, XRV and IBV (seasonal trends removed) were closely linked (*ρ* = 0.755; *p* < 0.001), indicating tight hydraulic coupling between these tissues. Furthermore, the daily amplitude of XRV was linearly and closely related to daily maximum T (*ρ* = 0.802; *p* < 0.001), mean daily solar radiation (*ρ* = 0.809; *p* < 0.001), and non-linearly related to daily maximum VPD (*R^2^* = 0.837; *p* < 0.001), indicating that the xylem of *Pinus sylvestris* reacts like a transpiration-driven passive hydraulic system.

## 1. Introduction

Apart from irreversible growth-related radius expansion, reversible daily and seasonal fluctuations in stem radius can be attributed to changes in stem hydration. These fluctuations occur mainly in the elastic tissues of the stem, i.e., parenchyma within the bark, phloem and cambium, e.g., refs. [1,2,3]. These tissues, along with sapwood, cell walls and needle mesophyll are important storage locations of water, which are required to counterbalance water deficits during periods of high transpiration (for a review see [4]). Due to the lignification of the cell walls in the xylem, which causes a reduced elasticity of this tissue, the xylem contracts less than the unlignified tissues of the inner bark (i.e., living phloem cells, parenchyma and the cambial zone) and therefore generally contributes only marginally to the reversible variations in the stem radius, e.g., refs. [3,5,6]. Other studies, however, found that xylem variations accounted for *c*. 30–50% of the total stem diameter changes [7,8], and in exceptional cases radial changes in the xylem may exceed those of the inner bark [9].

A close relationship found between xylem radial variations (XRV) and sap flow, e.g., refs. [5,10,11], indicates that the water tension in the conduits induced by transpiration leads to a contraction of the functional xylem tissue [12,13]. The tight hydraulic connections between the xylem and the water storage tissues of the inner bark via parenchyma cells (rays) subsequently lead to radial water transport between these tissues [14] causing diurnal changes in stem radius, i.e., shrinkage during the day and replenishment at night, in accordance with the water potential gradient within the stem [15,16,17]. The radial flow of water is assumed to be necessary to meet the water demand with increasing transpiration, preventing premature closure of the stomata and maintaining photosynthesis [4]. The time lag found in the stem diameter change of the inner bark compared to the xylem [8,13,16] is consistent with the view of a transpiration-driven passive hydraulic system, but was also related to higher osmotic concentrations in the phloem [18,19,20,21,22]. Osmotic adjustments have also been proposed to explain the findings that the width of the xylem shrinks while, concurrently, bark size expands, e.g., refs. [9,23,24].

Several authors observed that environmental factors affecting transpiration (e.g., vapour pressure deficit of the air; VPD) which lead to a corresponding change in stem water potential were closely linked with stem radius variations, e.g., refs. [25,26,27], while soil water content or precipitation showed a weaker relationship with changes in stem radius in *Pinus sylvestris* exposed to drought [27,28]. The greater significance of VPD than soil moisture for, e.g., vegetation growth and tree mortality has also been shown by several authors [29,30,31,32]. Regarding the influence of environmental factors on the diurnal shrinkage and swelling of the xylem, Offenthaler et al. [33] found that in Norway spruce (*Picea abies* [L.] Karst.), wood diameter decreased with increasing VPD, and periods of soil drought caused a successive decrease in pre-dawn xylem width. Sevanto et al. [34] reported that diurnal xylem diameter variations in *Pinus sylvestris* in southern Finland were most closely related to VPD and soil water content.

Combined measurements of the xylem and phloem radial variations by point dendrometers are valuable in order to study xylem–phloem interactions [6,8,17,20,35]. Simultaneous records of stem diameter variations are, however, rarely carried out [4], and even less so continuously over several years (but see [20]). Therefore, the main objectives of this study were to compare radial variation in the xylem and inner bark (IBV) of drought-stressed Scots pine (*Pinus sylvestris* L.) at daily and seasonal scales and to evaluate environmental drivers of XRV during the growing season. We expected that (i) on a daily scale, XRV precedes IBV and shows a significantly lower amplitude compared to IBV; (ii) on a seasonal scale, xylem radius decreases steadily during the period of the highest transpiration rates in summer; and (iii) XRV is controlled by atmospheric conditions, primarily VPD, rather than soil water availability.

## 2. Materials and Methods

### 2.1. Study Area

The selected study plot is situated in the montane belt (*c*. 750 m asl) within the inner Alpine dry valley of the Inn River (Tyrol, Austria, 47°13′53″ N, 10°50′51″ E). The plant community at the selected xeric site is an open Spring Heath-Pine wood (*Erico-Pinetum sylvestris*; [36]) with a canopy coverage of 75%. Long-term (1911–2018) mean annual air temperature and annual precipitation sum amount to 7.3 °C and 724 mm, respectively (meteorological station at Ötz, 812 m asl; 5 km from the study area). In the study years, the total annual precipitations were 941 mm (2019), 894 mm (2020) and 817 mm (2021).

According to [37], the soil type is a rendzic leptosol consisting of unconsolidated, coarse-textured material with low water-holding capacity, and shallow soils reach a maximum depth of 0.2 m. The study plot was slightly north-facing with a slope angle of 5°. The mean stem diameter of the selected trees (*n* = 4) amounted to 27 ± 2 cm (measured over bark at the height of dendrometer installation). Due to extreme environmental conditions prevailing within the study area, i.e., low water availability and nutrient deficiency of the substrate, the annual radial growth (mean ring widths *c*. 0.7 mm; [38]) and tree height of 6–7 m were very low for trees that were about 150 years old [39].

### 2.2. Microclimate Records

Climate conditions (air temperature, T_air_; relative air humidity, RH; precipitation, P), solar radiation (SR) and soil water content (SWC) were continuously recorded at the study plot from March 2019 through October 2021. T_air_, RH, SR and P sensors were mounted at 2 m aboveground (ONSET, Bourne, MA, USA). Soil moisture sensors (*n* = 3; Theta Probes Type ML2x, Delta-T, Cambridge, UK) were installed at 10 cm soil depth. The measuring interval for all the sensors was set to 30 min. The vapour pressure deficit of the air (VPD) was calculated based on half-hour records of T_air_ and RH according to the equation given in [40]. T_air_, RH, SR, SWC and VPD were expressed as daily means, while P was summed up to daily totals. 

### 2.3. Records of Inner Bark and Xylem Radial Variations

High-resolution changes in Scots pine whole stem (i.e., over living bark) and xylem radius were determined at 1 m stem height with automated point dendrometers (DR1, Ecomatik, Dachau, Germany). By recording stem radius variations over bark and on the xylem in parallel, xylem–phloem interactions can be analysed, e.g., [35]. To accomplish this, on four trees, one point dendrometer was mounted on the living phloem, i.e., the dead outer layers of the bark were removed to prevent the influence of hygroscopic shrinking and swelling of dead bark tissue on dendrometer traces [41]. To measure xylem radial variation (XRV), the sensor of the second dendrometer was positioned on a steel screw which was inserted *c*. 2–3 cm into the sapwood (Figure S1). In this way, the influences of resin production (sticking the sensor tip) and water losses through evaporation on dendrometer records were prevented. Variations in inner bark (IBVs), which included living phloem cells, the cambial zone and newly developed xylem and phloem tissue, were calculated by subtracting XRV from IBV. 

Point dendrometers were mounted on the north-facing side of the stem and at a distance of 10–15 cm in order to minimize the influence of solar heating and wound responses, respectively, on dendrometer traces. The effect of thermal expansion of the steel screw (13.9 × 10^−6^ °C^−1^), dendrometer sensor (0.2 μm °C^−1^) and of moist wood (7.9 × 10^−6^ °C^−1^; [34]) was eliminated by correcting the results on the basis of T_air_ recorded at the study plot. Hence, variations in the inner bark and xylem were corrected for thermal expansions by 0.2 μm °C^−1^ and 1.08 µm °C^−1^, respectively.

Daily variations in the inner bark and xylem were determined during three consecutive growing seasons (2019–2021), extending from April through September [42], by calculating the difference between the mean values of two consecutive days (‘‘daily mean approach’’, ref. [43]). While IBVs represent a combination of water- and growth-induced radius changes, XRVs represent water-induced changes caused by transpiration and the radial flow of water between the phloem and xylem, e.g., refs. [10,35,44]. Daily amplitudes of XRV were calculated by subtracting the daily minimum value from the daily maximum. Daily maximum and minimum values were extracted from 30 min records of XRV. The influences of environmental variables on XRV (daily xylem maximum, mean, minimum and amplitude) were calculated for the period April through September 2019–2021, i.e., the effects of stem frost on dendrometer records were avoided. Furthermore, time series of XRV and IBV were de-trended by subtracting a smoothed curve, which was based on a fast Fourier-transform low-pass filter (*n* = 10 points), from dendrometer records. The residuals calculated in this way were free of seasonal trends.

### 2.4. Data Analysis 

The Shapiro–Wilk test was applied to check for a normal distribution of dendrometer and environmental data. Due to the lack of normal distribution, Spearman correlation coefficients (*ρ*) were determined for linear relationships between environmental variables and XRV, and between residuals of XRV and IBV. Stepwise multiple regression analysis was used to identify the impact of environmental factors on daily XRV. All the statistical analyses were performed using the software packages STATISTICA (version 13.5.0.17; TIBCO Software Inc., Palo Alto, CA, USA) and SPSS (version 26.0.0.1; IBM SPSS Statistics for Windows; IBM Corp, Armonk, NY, USA).

## 3. Results

### 3.1. Environmental Conditions during the Growing Seasons 

**Figure 1:**
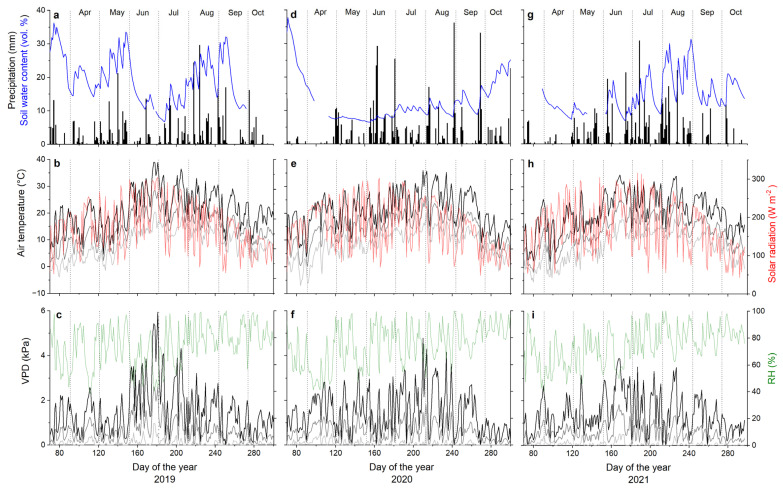
Climate variables and soil water content (Vol. %) recorded from early March through late October during 2019–2021 within the study plot. (**a**,**d**,**g**) Daily precipitation sum (bars) and soil water content in 10 cm soil depth (blue line). (**b**,**e**,**h**) Daily maximum (black line), mean (dark grey line) and minimum (light grey line) air temperature and daily mean solar radiation (red line). (**c**,**f**,**i**) Relative air humidity (thin green line) and maximum (black line), mean (dark grey line) and minimum (light grey line) vapour pressure deficit of the air. Missing soil water content data (blue lines in (**a**,**d**,**g**)) are due to logger failure.

The daily mean air temperature (T_air_) and maximum and minimum T_air_ recorded during the growing seasons (April–September) are depicted in Table 1. An exceptionally high T_air_ was recorded in late June 2019, reaching a maximum of 39 °C. Averaged over the growing season, VPD varied around 0.6 kPa (Table 1), approaching zero on rainy days and reaching a maximum of 5.95 kPa on 30 June 2019 (Figure 1). Total P during the study periods varied between 390 mm in 2019 and 461 mm in 2021 (Table 1). Depending on seasonal trends in P, SWC varied between a maximum of 33.5 Vol. % on 28 May 2019 and a minimum of 5.4 Vol. % on 3 June 2020 during dry periods with high T and VPD (Figure 1). Growing season daily mean SWC varied between 8.8 Vol. % in 2020 and 18.4 Vol. % in 2019 (Table 1). 

### 3.2. Radial Variations in Inner Bark and Xylem throughout the Study Period

**Figure 2:**
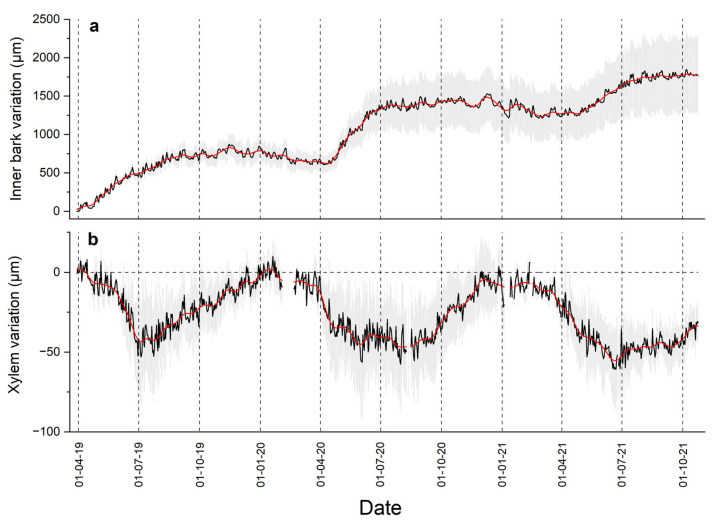
Inner bark (**a**) and xylem radial variations (**b**) from 1 April 2019 through 24 October 2021. Daily means ± standard error are shown in black and gray lines, respectively. Data were smoothed based on a fast Fourier-transform low-pass filter (red lines), whereby the number of points was set to 10 days. Missing data in (**b**) are due to logger failure.

Variations in the inner bark (IBV) recorded throughout the whole study period, i.e., April 2019–October 2021, are shown in Figure 2a, indicating that the duration of the radial growth phase lasted from early April throughout August/September. While radial growth reached *c*. 0.7 mm in 2019 and 2020, it decreased to *c*. 0.3 mm in 2021. With increasing T_air_ in April, the radial xylem width started to decrease, leading to a mean maximum radial shrinkage of *c*. 50 µm during the study years (Figure 2b). The swelling of the xylem started again in July/August with decreasing T_air_ and lasted until December, when the initial values were reached again. 

**Figure 3:**
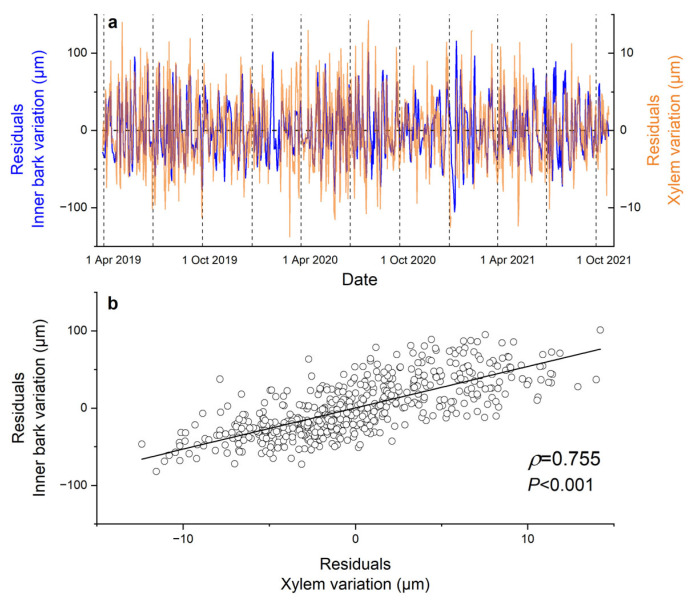
Residuals (**a**), i.e., dendrometer record minus smoothed curve (cf. Figure 2) of inner bark (blue line) and xylem radial variations (orange line) during the entire study period, and (**b**) their relationship during April through September 2019–2021 (*n* = 542). Spearman correlation coefficient (*ρ*) is indicated.

Residuals, i.e., deviations in inner bark and xylem from the smoothed long-term trend, fluctuated between ±100 µm and ±10 µm, respectively (Figure 3a). A close and highly significant direct relationship (Spearman correlation coefficient *ρ* = 0.755, *p* < 0.001) was detected between the residuals of IBV and XRV (Figure 3b).

### 3.3. Influence of Environmental Variables on Xylem Radial Variations

**Figure 4:**
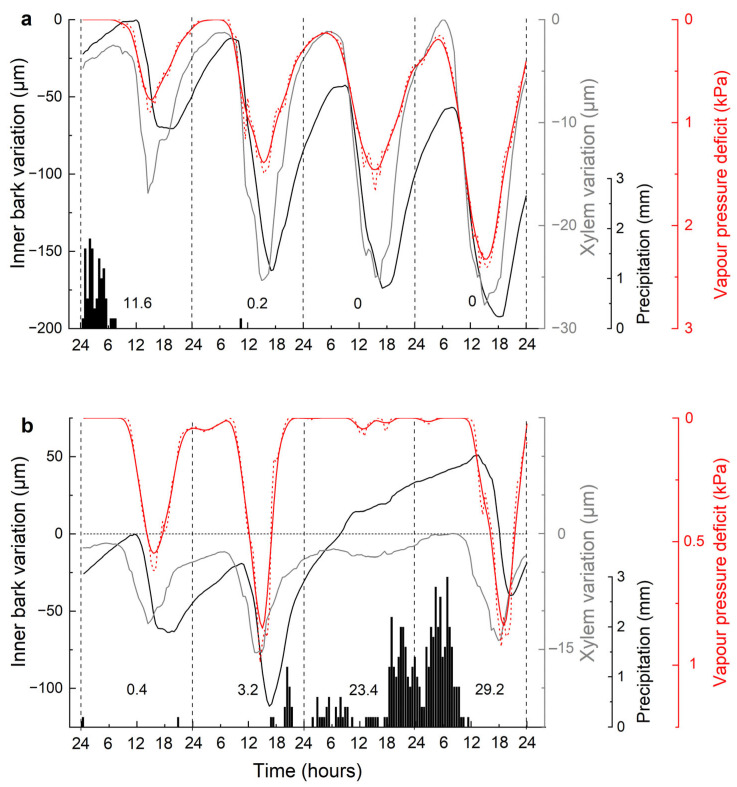
Daily cycle of inner bark (black line) and xylem radial variations (grey line) during a dry (**a**) and wet (**b**) period (dry and wet periods were 13–16 July 2019 and 8–11 June 2020, respectively). Both periods shown are within the growing season of *Pinus sylvestris* in the study area [42]. The zero points of inner bark and xylem variation were set to the highest value recorded on day 1 and during the four-day period, respectively. Half-hourly precipitation values are shown as bars and daily precipitation sums are given. Note inverted and different y-scales of vapour pressure deficit (red line) in (**a**,**b**). Vapour pressure deficit was smoothed using the LOWESS function.

**Figure 5:**
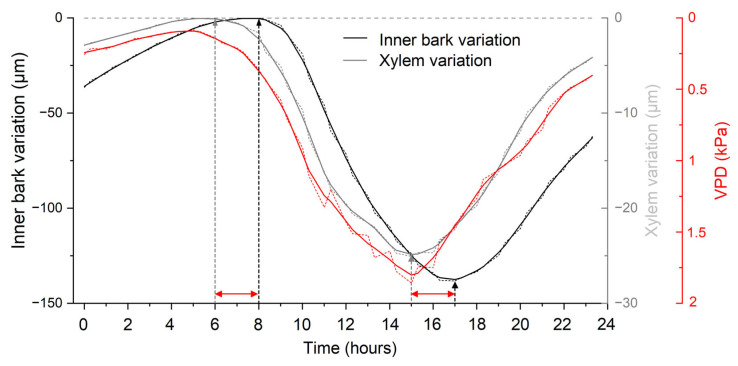
Mean values of daily inner bark (black line) and xylem radial variations (grey line) and vapour pressure deficit (VPD; red line) during the 3-day dry period (14–16 July 2019) depicted in Figure 4a. Note inverted y-scale of vapour pressure deficit. Red arrows show the time delay in the response of inner bark variation compared to xylem variation. All records were smoothed using the LOWESS function.

Daily cycles of IBV and XRV indicate a close relationship with VPD (Figure 4a,b). During exemplary dry (Figure 4a) and wet periods (Figure 4b), the shrinkage and swelling of the xylem started about 2 h earlier than that of the inner bark (Figure 5). During the dry period, xylem width recovered every day, while the inner bark increasingly shrank in radial thickness (Figure 4a). During a wet period in June (Figure 4b), the inner bark thickness not only recovered but, due to radial growth, increased by *c*. 50 µm, while the xylem width exceeded the previous days’ maximum by <5 µm. Daily maximum values of the xylem and inner bark were reached early in the morning around 6 a.m. and 8 a.m., respectively, and minimum values were recorded in the afternoon around 3 p.m. in xylem and around 5 p.m. in inner bark (Figure 5). The amplitude of daily variation during the dry period reached *c*. 140 µm in inner bark and *c*. 25 µm in xylem (Figure 4a and Figure 5).

During April through September, daily xylem radial variations (XRV) are negatively and highly significantly (*p* < 0.001) related to daily maximum, mean and minimum T_air_ along with daily maximum and mean VPD (Table 2). While the daily minimum XRV is most closely related (inverse relationship) to daily maximum T_air_ (*ρ* = −0.799) and VPD (*ρ* = −0.665), daily minimum XRV is directly related to daily minimum RH (*ρ* = 0.308) and daily P (*ρ* = 0.261). Finally, SWC is directly and most closely related to daily minimum XRV (*ρ* = 0.330; *p* < 0.001).

Using all the environmental variables listed in Table 2, multiple regression analyses revealed that the daily maximum, mean and minimum XRV were best predicted by the variables air temperature, air humidity and soil water content, with *R*^2^ ranging from 0.608 to 0.700 (Table 3).

**Figure 6:**
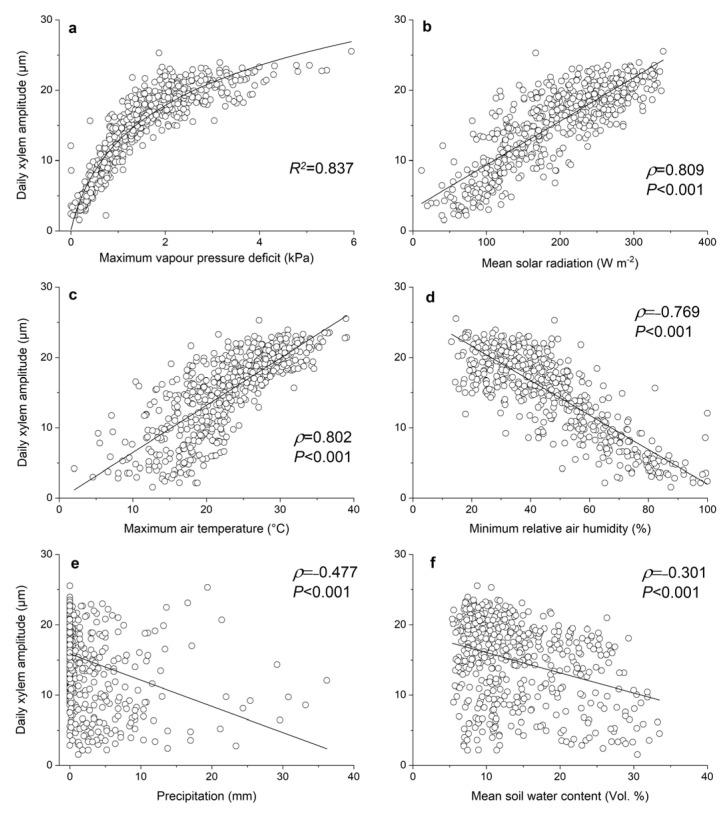
Relationships between daily amplitudes in radial xylem variations and daily environmental variables during April–September of 2019–2021 (*n* = 549, except for soil water content when *n* = 516). Data given in (**a**) were fit with a logarithmic function. Spearman correlation coefficients (*ρ*) and linear regression lines are shown in (**a**–**f**).

The environmental influences on daily xylem amplitude are depicted in Figure 6. The relationship between daily xylem amplitude and daily maximum VPD is non-linear (*R*^2^ = 0.837; Figure 6a), i.e., daily xylem amplitude remains largely stable when daily maximum VPD exceeds *c*. 3 kPa. Daily xylem amplitude is linearly and directly related to mean daily solar radiation (*ρ* = 0.809, *p* < 0.001; Figure 6b) and daily maximum T_air_ (*ρ* = 0.802, *p* < 0.001; Figure 6c). An inverse relationship exists between daily xylem amplitude and daily minimum RH (*ρ* = −0.769, *p* < 0.001; Figure 6d), P (*ρ* = −0.477, *p* < 0.001; Figure 6e) and SWC (*ρ* = −0.301, *p* < 0.001; Figure 6f). Spearman correlation coefficients for relationships between daily xylem amplitude and daily mean and minimum T_air_ amounted to *ρ* = 0.641 (*p* < 0.001) and *ρ* = 0.293 (*p* < 0.001), respectively. The influences of environmental conditions on the daily amplitude of inner bark are also significant (*p* < 0.001) but relationships are much less pronounced, e.g., daily maximum VPD, *ρ* = 0.523, daily maximum T_air_, ρ = 0.584, mean daily SR, *ρ* = 0.467, and daily minimum RH, *ρ* = −0.309.

**Figure 7:**
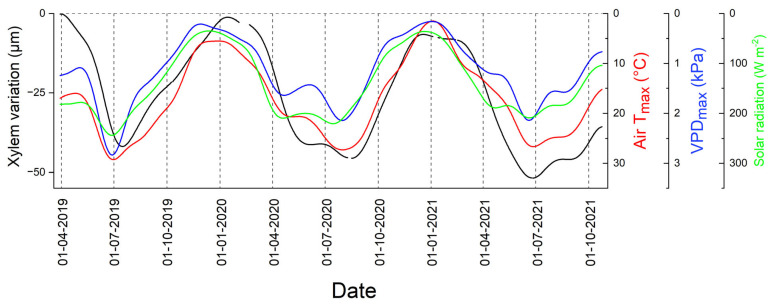
Xylem radial variations (black line), atmospheric parameters and solar radiation throughout the study period April 2019 through mid-October 2021 (*n* = 903). Daily maximum air temperature (Air T_max_), daily maximum vapour pressure deficit (VPD_max_) and mean daily solar radiation are indicated by red, blue and green lines, respectively (note inverted y-scales). All data were smoothed based on a fast Fourier-transform low-pass filter, whereby the number of points was set to 25 days.

In Figure 7, it is shown that environmental factors that influence plant transpiration (VPD, T_air_, SR) show a seasonal trend similar to XRV, whereby a lag effect in the response of XRV is apparent. Spearman correlations (*ρ*) between XRV and environmental variables throughout the entire study period (as depicted in Figure 7), which extended from April 2019 to mid-October 2021 (*n* = 903), amount to −0.827, −0.755 and −0.650 for daily maximum T_air_, daily maximum VPD and mean daily SR, respectively (all *p* < 0.001).

## 4. Discussion

### 4.1. Hydraulic Coupling between Phloem and Xylem

In our study, we determined the daily and seasonal change in XRV throughout three growing seasons in mature, *c*. 150 years old, *Pinus sylvestris* in a drought-prone environment [37,42,45]. XRV amounted to *c*. 10 and 50 µm on daily and seasonal scales, respectively. Removing the seasonal trend in the radial variation of the xylem and phloem revealed that the daily amplitude of XRV was about 1/10 that of IBV, confirming our expectation. The rigid lignified cell walls of dead xylem cells prevent distinct radial changes, which can occur in the much more elastic non-lignified living phloem cells, the cambial zone and enlarging xylem cells. This explains the almost ten times higher diurnal radial variations found in the latter tissue. The pronounced different contribution of these water storage tissues to stem diameter changes was also demonstrated in numerous studies, e.g., [8,9,22,46]. 

The significant (*p* < 0.001) linear relationship between the residuals of XRV and IBV indicates a tight hydraulic coupling between the xylem and phloem. Water exchange is caused by differences in the water potential of these closely connected tissues and can take place in both directions, depending on the actual gradient in the water potential, e.g., [14,22,35,47]. Several authors reported a change in osmolality in the phloem of evergreen tree species to reduce water loss in the phloem, especially when atmospheric water demand is high, e.g., [8,9,14,17]. We did not, however, find an opposite response of XRV and IBV, i.e., shrinkage of the xylem concurrent with expansion of the phloem, and therefore there are no indications for osmotic adjustments in *Pinus sylvestris* at 1 m stem height during our study period of three growing seasons. This interpretation is supported by findings of Lazzarin et al. [22], who detected strong indications of osmoregulation at the twig level, but not at the stem base, in mature *Pinus sylvestris* at a comparable drought-exposed site.

### 4.2. Environmental Factors as Determinants of Xylem Radial Variation 

Although the study area is prone to dry conditions and precipitation limits radial stem growth and xylem formation [38,45], XRV is more closely related to evaporative demand (VPD, SR and T_air_) than to water availability (precipitation and SWC). The close relationships found among XRV and VPD, air temperature, RH and solar radiation can be attributed to the influence of these environmental factors on transpiration. This assumption is confirmed by the finding that transpiration is closely coupled to sap flow [48,49] and xylem water potential [50,51]. Non-linearity between VPD and the daily amplitude of XRV is most likely caused by the rigidity of the lignified cell walls of the xylem, which are becoming increasingly resistant to shrinkage as VPD rises. The tendency of XRV to saturate at high VPD might also be caused by a decrease in canopy conductance with increasing VPD [49,52], which prevents the further lowering of the water potential in the xylem. 

Daily stem radius variations and stem water deficit, i.e., stem radius variations detrended for growth [53] of conifers exposed to soil dryness, were also found to be more strongly controlled by atmospheric conditions (VPD, air temperature, RH) than by precipitation or SWC [45,54]. This finding confirms our hypothesis that XRV is primarily related to environmental variables which influence transpiration. The lack of close relationships between precipitation and SWC and XRV indicates that transpiration uses water stored in the trunk rather than soil water, e.g., [55,56]. The fact that VPD restricts tree growth and leads to hydraulic damage before soil moisture becomes a limiting factor was also reported by several authors [30,32,57,58]. Because the water uptake of *Pinus sylvestris* in the shallow and stony soils prevailing within the study area may also occur from deeper soil layers, the results indicate that stem water status may differ from that determined in the upper soil layers (5–15 cm soil depth), which is consistent with the findings of several authors [59,60,61].

### 4.3. Daily and Seasonal Xylem Variations

The diel cycles of VPD, temperature and solar radiation affect transpiration, which induces cyclic changes in stem water potential and, as a consequence, in the radial thickness of the xylem and the phloem [4,48]. The strong influence of VPD on XRV is indicated by the daily and seasonal minimum of XRV which was recorded around 3 p.m. and in late June through to the end of July, respectively, at the time when maximum daily and seasonal VPD was reached. The earlier shrinkage and swelling of the xylem in daily cycles compared to inner bark (time lag amounting to about 2–3 h) is explained by transpiration causing tension in the xylem, followed by radial water flow from the inner bark to the xylem [14,16] due to the transpiration-driven passive hydraulic system. The swelling of the xylem radius (followed by an increase in stem diameter) during the daily cycle is related to water uptake from the soil and water flow from the xylem to phloem caused by the higher sugar content in the latter (osmotic water transport). In accordance with our hypothesis and with several other studies [4,6,13,16], radial variations in the inner bark followed behind the radial variations in the xylem. Sevanto et al. [8,18] suggests that this time lag reflects resistance in the hydraulic connection between storage compartments (bark, phloem, cambium) and the xylem and/or changes in turgor pressure in the phloem, e.g., low osmotic potential in the phloem caused by high sugar content increases the time lag between the radial shrinkage of the xylem and inner bark. 

As expected, seasonal changes in XRV indicate that increasing evaporative demand during the growing season leads to the successive shrinkage of the xylem. Only reduced transpiration rates under conditions of low evaporative demand, i.e., low temperature, VPD and SR, allow the replenishment of water reservoirs in the xylem via continuous water uptake from the soil, leading to an increase in xylem radial thickness to the level of the initial values.

## 5. Conclusions

The results of our study provided an insight into the environmental control of XRV in drought-exposed mature *Pinus sylvestris* on a daily and seasonal scale. The close coupling of XRV (this study) and stem water deficit [55] to atmospheric conditions (especially VPD) within the study area points to the strong dependence of stem water status on changes in these variables. Because atmospheric water demand increases exponentially with an increase in temperature [62], and thus causes the water content in the stem to decrease, especially under drought, tree growth, hydraulic functionality and mortality will be increasingly affected with ongoing climate warming, e.g., [31,63,64,65].

## Figures and Tables

**Table 1:** Climatic parameters for the growing seasons (April through September) 2019–2021. Maximum values are given in brackets (P = precipitation, SWC = soil water content, T_air_ = air temperature, VPD = vapour pressure deficit of the air).

Year	T_air_ (°C)	VPD (kPa)	P (mm)	SWC (Vol. %)
2019	15.6 (39.0)	0.65 (5.95)	390 (30)	18.4 (33.5)
2020	15.8 (36.0)	0.63 (4.78)	461 (36)	8.8 (16.1)
2021	14.9 (34.4)	0.58 (3.90)	430 (31)	14.4 (31.3)

**Table 2:** Spearman correlation coefficients (ρ) between environmental variables and daily radial xylem variations (Xylmax, Xylmean, Xylmin = daily maximum, mean and minimum radial xylem variations, respectively) during April through September of 2019–2021. Number of samples (*n*) are 549 except for soil water content when *n* = 516 (max, mean, min = daily maximum, mean, and minimum values of environmental variables; Air T = air temperature; VPD = vapour pressure deficit of the air; RH = relative air humidity; P = precipitation; SWC = volumetric soil water content). * = *p* < 0.05; ** = *p* < 0.01; *** = *p* < 0.001.

	Air Tmax (°C)	Air Tmean (°C)	Air Tmin (°C)	VPDmax (kPa)	VPDmean (kPa)	VPDmin (kPa)
Xylmax	−0.558 ***	−0.656 ***	−0.667 ***	−0.325 ***	−0.234 ***	0.030
Xylmean	−0.691 ***	−0.746 ***	−0.661 ***	−0.508 ***	−0.423 ***	−0.108 *
Xylmin	−0.799 ***	−0.795 ***	−0.637 ***	−0.665 ***	−0.569 ***	−0.193 ***
	**RHmax** **(%)**	**Rhmean (%)**	**Rhmin (%)**	**P** **(mm)**	**SWC** **(%)**	
Xylmax	−0.112 **	−0.084 *	−0.047	0.054	0.263 ***	
Xylmean	0.026	0.105 *	0.146 ***	0.164 ***	0.272 ***	
Xylmin	0.115 **	0.245 ***	0.308 ***	0.261 ***	0.330 ***	

**Table 3:** Stepwise multiple regression models showing effects of environmental variables on daily radial xylem variation. Non-standardized regression coefficients are shown. Standard errors are given in parentheses (Xyl_max_, Xyl_mean_, Xyl_min_ = daily maximum, mean, and minimum radial xylem variations, respectively; T_air_ = air temperature, RH = relative air humidity, SWC = volumetric soil water content). ** = *p* < 0.01; *** = *p* < 0.001.

Dependent Variable	Constant	T_air_	RH	SWC	*R*^2^ Adjusted	F-Value (3, 512)
Xyl_max_	18.664 (1.915)	−1.513 ***^1^ (0.056)	−0.234 ***^1^ (0.020)	0.220 *** (0.044)	0.613	273.48
Xyl_mean_	26.220 (4.170)	−1.849 ***^1^ (0.068)	−0.210 ***^2^ (0.041)	0.161 ** (0.053)	0.608	267.47
Xyl_min_	45.253 (4.226)	−1.656 ***^2^ (0.051)	−0.323 ***^2^ (0.040)	0.316 *** (0.051)	0.700	401.36

Notes: ^1^—daily mean values; ^2^—daily maximum values.

## Data Availability

The data presented in this study are openly available on Zenodo at 10.5281/zenodo.8202893 (accessed on 1 August 2023).

## Supplementary Materials

The following supporting information can be downloaded at: https://www.mdpi.com/article/10.3390/biology12091251/s1. Figure S1: Photographs showing the mounting of point dendrometers on the bark and on the xylem.
