# Supplementary material for: Seasonal and Daily Xylem Radius Variations in Scots Pine Are Closely Linked to Environmental Factors Affecting Transpiration
Source: Biology (Basel). 2023 Sep 18;12(9):1251. doi: 10.3390/biology12091251 (PMC10525319; doi:10.3390/biology12091251)
Supplement: Supplementary file 1 [file biology-12-01251-s001.zip › biology-2597317-supplementary.pdf]

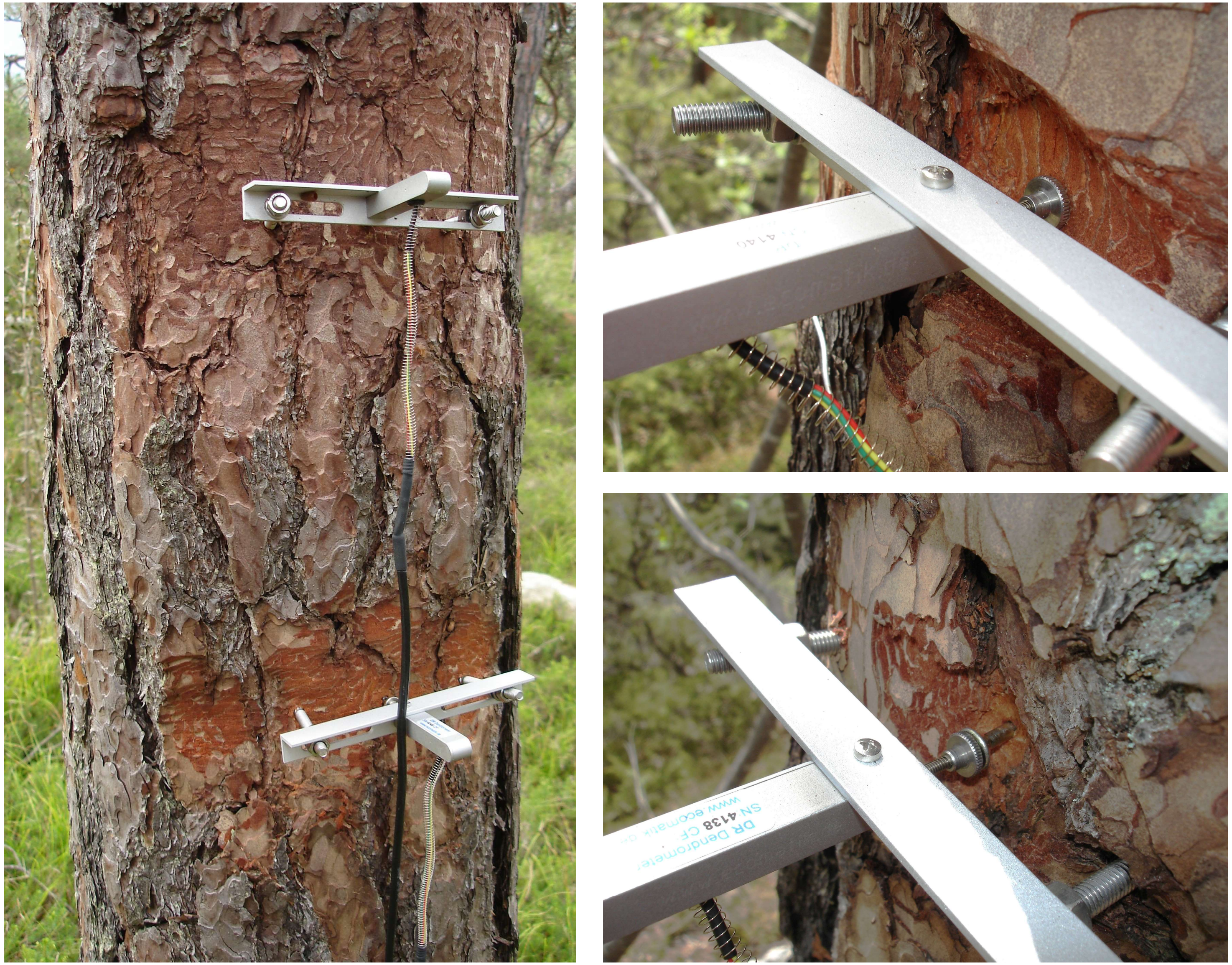

Figure S1. Overview (left) and close-up (right) of point dendrometers mounted on the bark (top; dead outer bark layers were removed) and on the xylem (bottom; sensor positioned on a screw, which was inserted *c*. 2–3 cm into the sapwood) of a sample tree (*Pinus sylvestris* L.).
